# Physiological Roles and Mechanisms of Action of Class I TCP Transcription Factors

**DOI:** 10.3390/ijms24065437

**Published:** 2023-03-12

**Authors:** Ivana L. Viola, Antonela L. Alem, Rocío M. Jure, Daniel H. Gonzalez

**Affiliations:** Instituto de Agrobiotecnología del Litoral (CONICET-UNL), Facultad de Bioquímica y Ciencias Biológicas, Universidad Nacional del Litoral, Santa Fe 3000, Argentina

**Keywords:** transcription factor, TCP family, plant development, transcriptional regulation, postranslational regulation

## Abstract

TEOSINTE BRANCHED1, CYCLOIDEA, PROLIFERATING CELL FACTOR 1 and 2 (TCP) proteins constitute a plant-specific transcription factors family exerting effects on multiple aspects of plant development, such as germination, embryogenesis, leaf and flower morphogenesis, and pollen development, through the recruitment of other factors and the modulation of different hormonal pathways. They are divided into two main classes, I and II. This review focuses on the function and regulation of class I TCP proteins (TCPs). We describe the role of class I TCPs in cell growth and proliferation and summarize recent progresses in understanding the function of class I TCPs in diverse developmental processes, defense, and abiotic stress responses. In addition, their function in redox signaling and the interplay between class I TCPs and proteins involved in immunity and transcriptional and posttranslational regulation is discussed.

## 1. Introduction

**Figure 1:**
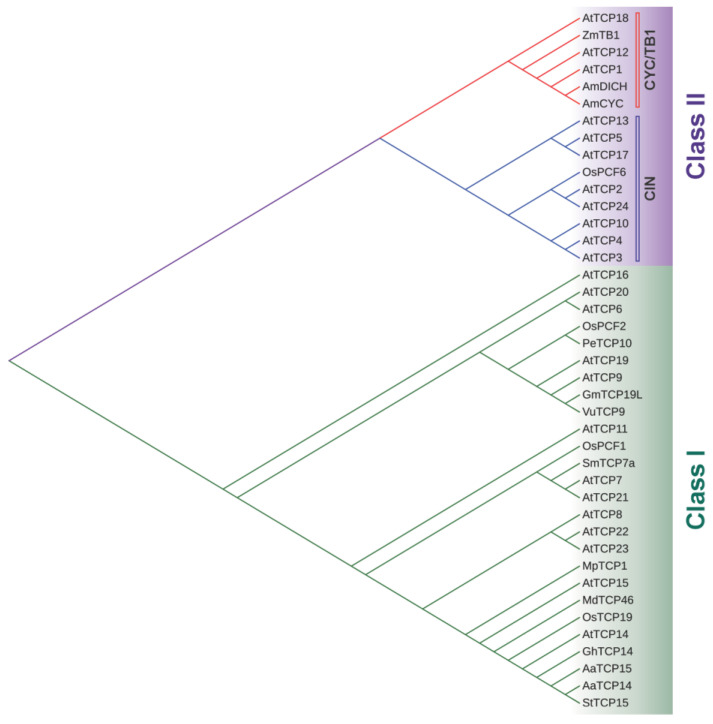
Phylogenetic tree of the TCP family showing the different classes (I and II) and clades (CYC/TB1 and CIN). Amino acid sequences of TCP proteins from *Arabidopsis thaliana*, the four founding members of the TCP family (ZmTB1, AmCYC, OsPCF1 and OsPCF2) and the other TCPs mentioned throughout this review were aligned with Clustal Omega (https://www.ebi.ac.uk/Tools/msa/clustalo/, accessed on 10 February 2023) and used to construct the tree using the Neighbor-Joining method (https://www.ebi.ac.uk/Tools/phylogeny/simple_phylogeny/, accessed on 10 February 2023). The tree was displayed using iTOL (https://itol.embl.de/itol.cgi, accessed on 10 February 2023). At, *Arabidopsis thaliana*; Am, *Antirrhinum majus*; Zm, *Zea mays*; Os, *Oryza sativa*; Gh, *Gossypium hirsutum*; Aa, *Artemisia annua*; Gm, *Glycine max*; Sm, *Solanum melongena*; Pe, *Phyllostachys heterocycle*; St, *Solanum tuberosum*; Vu, *Vigna unguiculata*; Mp, *Marchantia polymorpha*.

The TCP gene family encodes a plant-specific transcription factor family named after the first described members: TEOSINTE BRANCHED1 (TB1) from maize (*Zea mays*), CYCLOIDEA (CYC) from snapdragon (*Antirrhinum majus*), and PCF1 and PCF2 from rice (*Oryza sativa*) [1]. *TB1* is involved in the control of axillary meristems in maize [2], *CYC* affects snapdragon flower morphology [3], and PCF1/2 binds to the promoter of the *proliferating cell nuclear antigen* (*PCNA*) gene in rice [4]. In 1999, Cubas et al. determined that these proteins share a conserved domain, named TCP, involved in the interaction with DNA and the formation of dimers, and they divided them into two classes, I (or PCF) and II (or CYC/TB1) (Figure 1). The TCP domain contains a basic region followed by two alpha helices connected with a loop, which is reminiscent of the bHLH domain present in a different family of transcription factors. However, the presence of helix-breaking amino acids in the basic region indicated that the TCP domain is a novel DNA-binding domain exclusive to plants. The difference between both classes lies in features located both within and outside the TCP domain. Within the TCP domain, each subfamily differs in the length of the basic region, the composition of their bipartite nuclear localization signal (NLS), the residue composition of the loop and hydrophilic faces of the helices, and the length of helix II [1]. Outside the TCP domain, class I TCPs have short regions flanking the domain while most of the class II TCPs have an arginine-rich domain or R domain [1] and an ECE motif (glutamic acid-cysteine-glutamic acid) between the TCP and R domains [5]. Class II TCPs are further divided into two clades: CYC/TB1 (or ECE) and CIN (*CINCINNATA*-like) [6]. In *Arabidopsis*, the TCP family is formed by 24 members distributed in all chromosomes, 13 from class I: TCP6, TCP7, TCP8, TCP9, TCP11,TCP14, TCP15, TCP16, TCP19, TCP20, TCP21, TCP22, TCP23, and 11 from class II: TCP2, TCP3, TCP4, TCP5, TCP10, TCP13, TCP17, TCP24 from the CIN clade and TCP1, TCP12/BRC2, TCP18/BRC1 from the CYC/TB1 clade [1,7] (Figure 1). Over the years, several studies have been made to analyze the structures and consensus DNA-binding sequences of the TCP domain. Class I TCP proteins recognize the consensus binding sequence GGNCCCAC, whereas class II proteins prefer the rather similar sequence GGGNCCAC and the different specificity of both classes has been attributed to changes in the identity of a specific residue located in the basic region [8,9,10]. In 2020, the crystal structure of the TCP domain was finally elucidated. Sun et al. [11] determined that the TCP domain of a class II TCP from *Oryza sativa*, OsPCF6, forms a homodimer in which each monomer folds into a ribbon-helix-helix (RHH) type structure, similar to the motif found in the RHH superfamily of transcription factors, despite differences in their sequence. Recently, the crystallization of the DNA complexes of both class I and class II TCP domains revealed that the TCP domain defines a distinct DNA recognition module with a unique binding mechanism. TCP domain homodimers adopt a three-site recognition mode, binding DNA through a central pair of β-strands formed in the dimer interface and two basic flexible loops from the N-terminus of each monomer. This allows the TCP domain to display broad specificity for a range of DNA sequences [12]. Besides the TCP motif, numerous intrinsically disordered regions (IDRs) have been found using bioinformatic analysis, which explains the impossibility to crystallize the complete protein [13]. In general, all TCPs seem to contain long regions with consecutive disordered residues distributed throughout their sequence, with class I TCPs presenting greater disorder than class II [13,14]. For example, the class I TCP from *Arabidopsis* TCP8 is predicted to have three IDRs throughout its sequence [13]. Homo- or heterodimerization or oligomerization of TCPs is necessary to bind DNA and protein-protein interactions have been described between TCP proteins, with a preference for binding members from their own TCP class [8,15,16]. In addition, interactions with non-TCP proteins affect TCP functions [17] providing a means to modulate their activity in different developmental stages.

Although a common ancestor of TCP genes has not been identified, TCP family members are ancient proteins since they were found in species such as the green algae *Cosmarium*, the moss *Physcomitrella patens*, the lycophyte *Selaginella* [7], and the early-diverging land plant *Marchantia polymorpha* [18]. However, they are not present in the unicellular algae *Chlamydomonas* [6]. Besides *Arabidopsis*, TCPs were characterized in several land plants, as rice, tomato, soybean, cotton, chrysanthemum, peach, apple, *Setaria italica* and *Setaria viridis* [19,20,21,22,23,24,25,26,27,28,29], among others. In addition, genome-wide analysis allowed the identification of TCP genes in numerous species of commercial interest, as tobacco, banana, *Brassica napus*, cucumber, watermelon, strawberry, eggplant, *Ginkgo biloba*, and tea [22,30,31,32,33,34,35,36,37,38]. For the interested reader, an exhaustive analysis of the phylogeny and the evolution of the TCP family in 37 plant species from lower to higher plants was recently reported by Zhou et al. [39].

Although at first TCPs were known as modulators of cell elongation and division processes, today we know that they are involved in a wide range of biological processes throughout the entire life of plants, acting as crucial regulators of responses to internal and external signals through the recruitment of other proteins and the modulation of hormonal signaling pathways [16,40]. The TCPs from class II have been studied in several species and various reviews have been recently published [41,42,43,44], whereas class I TCPs were mainly characterized in *Arabidopsis thaliana*. In this review, we will examine the roles of class I TCPs in plant growth and development, as well as the modulation of their activity through interaction with other proteins and redox interconversions, proteolytic processing, or intra- or intercellular movement. Additionally, we will review the function of these proteins in response to different environmental conditions.

## 2. Biological Processes Modulated by Class I TCPs

### 2.1. Role of Class I TCPs in Cell Division, Growth and Expansion

The growth of organs is due to processes of cell proliferation and cell enlargement. Leaves differentiate from the borders of the shoot apical meristem as a group of cells that undergo active proliferation and progressively enter into a growth phase in which cell division is arrested while DNA replication persists [45,46]. This process, known as endoreplication, increases the ploidy level of cells, which is usually related to their size [45,47]. Finally, cells increase their volume by expansion, which implies loosening of the cell wall, uptake of water and vacuole enlargement. Roots also grow through processes of cell proliferation and enlargement, which take place at different distances from the root tip, where the meristem is located. Other organs, like hypocotyls and cotyledons, grow mainly by cell expansion.

**Figure 2:**
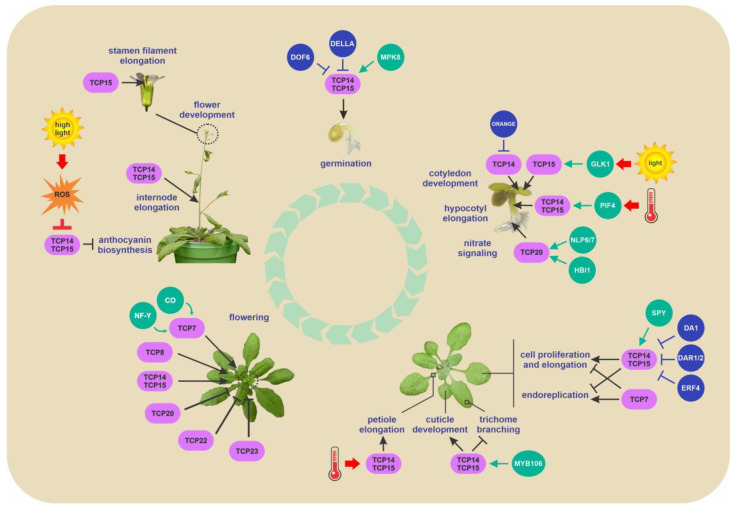
Scheme of the growth and developmental processes regulated by class I TCPs in *Arabidopsis*. The environmental conditions and interacting proteins that regulate class I TCP activity or stability are indicated. TCP-interacting proteins are shown as spheres. Green arrows and blue T-shaped lines indicate promotion and inhibition of TCP activity or stability, respectively. Red arrows and T-shaped lines indicate stimulatory and inhibitory effects by exogenous factors, respectively. The positive and negative regulatory actions of class I TCP proteins in biological processes are indicated by black arrows and T-shaped lines, respectively. For the purposes of illustration, some TCP-modulated processes are shown in one life stage, but may be operative also in other stages; see text for details.

Initial characterization of plants with altered TCP function related them to the control of cell proliferation [1]. However, these studies were performed with members of the class II, for which mutants with evident phenotypic alterations were available. Later on, more detailed studies on the function of class I TCPs showed that members of this class also have a role in this process, since higher order mutants, overexpressing plants and plants that express dominant repressor forms (TCP-SRDX) of class I TCPs show an altered expression of cell cycle-related genes, like *CYCA1;1*, *CYCA2;3*, *CYCB1;1*, *CYCB1;2*, *CYCD1;1*, *CYCD3;1*, *CDC20*, *PCNA1*, *PCNA2*, *RBR1*, *E2FB*, *MCM2*, *MCM3*, *MCM7*, *CDT1a*, *POLA2*, *WEE1*, and *FZR2* [48,49,50,51,52,53,54,55,56,57]. While the existence of a general role of class I TCPs in the regulation of cell cycle genes and cell proliferation seems clear, the specific effects of different TCPs seem to vary. Single and/or double mutants in the related class I *Arabidopsis* genes *TCP14* and *TCP15* show decreased expression of cell cycle genes [48,51,52,55,56,57], suggesting that these TCPs positively affect cell proliferation. In agreement with this, these mutants show increased endoreplication, observable mainly in trichome cells, which are overbranched and contain more DNA [57]. A role of TCP14 and TCP15 in regulating cell proliferation and endoreplication also in leaf cells other than trichomes was suggested by studies on the function of the ubiquitin receptor DA1 and DA1-related (DAR) proteins, which restrict cell proliferation and promote endoreplication [52]. It was observed that loss-of-function of *TCP14* and *TCP15* in a *da1 dar1 dar2* mutant background increases leaf cell size and nuclear ploidy [52]. It was proposed that DA1 and DAR proteins affect cell proliferation and endoreplication acting on the stability of TCP14 and TCP15 and that the stabilization of the class I TCPs in the *da1 dar1 dar2* mutant background is responsible for the decreased nuclear ploidy levels observed in the leaves of this mutant. The effect of the mutation of *TCP14* and *TCP15* in a wild-type background may not be evident in leaf cells other than trichomes due to functional redundancy with other TCPs. This is also suggested by the fact that plants that express TCP15-SRDX from the *TCP15* promoter in a wild-type background show increased cell size and DNA content in leaves, as well as overbranched trichomes [49]. The expression of this dominant repressor form most likely affects the function of endogenous TCP15 and of other related TCPs, thus revealing phenotypic changes that may be obscured by genetic redundancy [58]. The fact that an increase in nuclear ploidy is observed in a *tcp8 tcp14 tcp15* triple mutant [54] suggests that TCP8 may be one of the class I proteins that negatively regulate endoreplication together with TCP14 and TCP15 in leaves. It must also be noted that some studies reported that leaves from plants that express TCP14-SRDX or TCP15-SRDX contain smaller cells, which is contrary to the report mentioned above [48,59]. The reason for this discrepancy is not evident, but may be related to differences in expression levels and/or domains of the TCP-SRDX proteins, or even to the developmental stages or leaf sectors in which the cell sizes were analyzed. One possibility is that the respective dominant repressor forms used in each case affected the function of different class I TCPs with positive and negative functions in cell proliferation. Related to this, the quintuple mutant *tcp8 15 21 22 23* and the septuple mutant *tcp7 8 14 15 21 22 23* show increased expression of cell cycle promoting genes and decreased nuclear ploidy, respectively [50,54], which is contrary to observations made with the mutant in *TCP8*, *TCP14* and *TCP15* and indicates that TCP7, TCP21, TCP22 and/or TCP23 may be negative regulators of cell proliferation. In agreement, a mutant named *lcu*, in which TCP7 is transformed into a repressor, shows decreased leaf cell size and nuclear ploidy, also revealing TCP7 as a possible promoter of endoreplication through negative regulation of cell proliferation [54]. Clearly, a complex situation in which different class I TCPs have opposing roles in influencing cell cycle progression emerges from the results described above. The opposing effects of different class I TCPs on cell proliferation and endoreplication are probably related to the activation of different target genes. For example, TCP7 induces the expression of the cyclin *CYCD1;1* gene, whose overexpression promotes endoreplication in *Arabidopsis* [54], while TCP14 and TCP15 seem to induce mainly genes involved in the promotion of cell proliferation [48,49,51,52,53,57] (Figure 2). In addition, the fact that many of the class I TCPs with opposing functions are able to interact [15], suggests that they may interfere with each other by forming heterodimers. Virtually nothing is known about the formation of these heterodimers in plant cells and how this affects the function of the different members of the family. To add complexity, class I TCPs that have opposing roles in cell proliferation, like TCP7, TCP14 and TCP15, seem to have similar roles in other processes, like the regulation of flowering time [60,61]. Different protein-protein interactions and/or post-translational modifications may be at the basis of the functional diversity evidenced by different class I TCPs.

There is also evidence that class I TCPs TCP14 and TCP15 positively influence cell expansion in several organs, like hypocotyls, cotyledons, stamen filaments and germinating seeds [55,62,63,64,65,66,67]. In most of these processes, class I TCPs are mediators of the effect of gibberellin (GA) on growth, since they are inhibited through protein-protein interactions with DELLA proteins [51,56], negative modulators of GA responses [68]. A common theme in TCP action is the direct activation of genes from the *SAUR* family, involved in transducing the effects of auxin and other growth hormones to promote cell wall acidification and loosening [69]. In hypocotyls and cotyledons, the TCPs mediate the response to environmental factors, like temperature or illumination conditions, into growth responses through interactions with transcription factors involved in the respective processes [62,66] (Figure 2, Table S1). Apparently, these interactions recruit the TCPs to different gene promoters, thus exerting specific effects in each case. In addition to *SAUR* genes, the TCPs also induce the expression of other genes related to cell expansion, like GA synthesis and *EXPANSIN* genes [55,62,65]. Mutants in class I *TCP* genes also show defective growth responses to auxin, which is probably related to their direct role in the regulation of a subset of auxin responsive genes [63].

In summary, class I TCPs influence different aspects of cellular processes related to organ growth, like cell proliferation, endoreplication and cell expansion. Since, especially in leaves, these processes take place in an ordered fashion at defined temporal and spatial windows, how the TCPs are engaged into them at the correct time and place is a question that deserves investigation to fully understand their mechanism of action.

### 2.2. Role of Class I TCPs in Germination

Control of the switch from seed dormancy to germination is key to successful plant reproduction and establishment. Dormancy is a physiological mechanism that blocks seed germination under unfavorable conditions, which means that environmental signals such as temperature, light quality and others influence dormancy release [70]. At the same time, hormones actively participate in this process. Germination of mature seeds is inhibited by abscisic acid (ABA), while favorable environmental conditions promote GA biosynthesis and reduce ABA levels. The balance between these two antagonistic hormones promotes either dormancy or germination.

A series of studies indicated that TCP14 and TCP15 mediate the promotion of seed germination by GA in *Arabidopsis* [55,56,65,71]. These two TCPs are expressed in developing embryos and in seeds during germination and are necessary for root emergence [56,71]. Mutants in *TCP14* and/or *TCP15* show increased sensitivity to ABA and the GA biosynthesis inhibitor paclobutrazol and decreased sensitivity to GA during germination [56,71]. It is widely established that GAs regulate cell elongation and cell division by promoting the degradation of the DELLA proteins. TCP14 and TCP15 act downstream of GA and the stratification pathways which promote germination [56]. A molecular mechanism explaining the action of DELLAs on seed germination has been proposed by Resentini et al. [56]; DELLAs inhibit TCP14 and TCP15 activities upon interacting with them, which restricts cell-cycle progression in the embryonic root apical meristem in order to maintain the embryo in a quiescent state (Figure 2, Table S1). The DELLA-TCP module acts as a relay for environmental information into the cell cycle at the root apical meristem to coordinate root emergence with other events during seed germination [56]. Consistent with the description of these transcription factors as promoters of embryo growth potential, it has been found that TCP14 directly binds to the expansin *EXPA9* gene and promotes its expression [65], which results in the induction of cell expansion. Consequently, the reduction of germination efficiency observed in the *tcp14 tcp15* mutant is at least partially explained by a decrease in *EXPA9* expression, as supported by the partial rescue of this phenotype under GA-limiting conditions by overexpression of this gene [65].

Other reports associated TCP14 with additional partners during germination (Figure 2, Table S1). For example, TCP14 interacts with the DNA BINDING WITH ONE FINGER 6 (DOF6) transcription factor, a repressor of seed germination. This avoids the activation of the ABA biosynthetic gene *ABA DEFICIENT1* (*ABA1*) and other ABA-related stress genes [72], allowing TCP14 to promote cell proliferation and germination. Additionally, TCP14 regulates the dormancy-to-germination transition together with the MAP kinase MPK8 [55]. MPK8 specifically interacts with TCP14 in vivo and both proteins operate in a common pathway controlling genes related to seed maturation, cell expansion, cell proliferation, DNA replication, and cell cycle genes [55]. MPK8 is able to phosphorylate TCP14 in vitro, but the role of this modification in vivo is unknown. For instance, MPK8 is able to stimulate the transcriptional activity of TCP14 but phosphorylation does not seem to be required in this case [55]. Moreover, *mpk8* and *tcp14* mutant seeds show a significant reduction in the response to GA, highlighting their role in transducing the effect of this hormone [55]. Further studies are needed to better understand the relationship of ABA with the MPK8-TCP14 pathway and possible interconnections with the transcription factor DOF6.

### 2.3. Role of Class I TCPs in Epidermis Development

The aerial epidermal layer of plants is formed by different types of specialized cells like pavement cells, trichomes, and stomata guard cells and is covered by the cuticle, a hydrophobic layer composed of cutin and waxes [73]. As the interface between the plant and the environment, this tissue is involved in several processes such as osmotic regulation, defense against herbivore attack, and protection from excess transpiration and ultraviolet light [74,75,76]. Trichomes are epidermal outgrowths that function in the protection of plants from different biotic and abiotic challenges. In *Arabidopsis*, trichomes are branched single cells found in leaves, stems, and sepals with different morphology and density in each organ. Trichome branching is a consequence of progenitor cells switching to endoreplication and changing the cell ploidy, which affects trichome size and branch number [77]. As mentioned before, TCP14 and TCP15 have been reported to negatively regulate endoreplication [48,49,51,52,53,57,78], for instance, by directly activating *CYCA2;3* and *RBR* expression, two cell cycle genes that act as endoreplication negative regulators, in leaves [49] and inflorescence shoot apices [51]. In fact, *Arabidopsis* plants expressing the repressive forms TCP14-SRDX or TCP15-SRDX, as well as the *tcp14-6* and *tcp15-3* single and double mutants, present overbranched trichomes [48,49,57]. In addition to regulating cell cycle genes, TCP15 directly activates *MYB106*, which encodes a MIXTA-like transcription factor involved in the regulation of cuticle biosynthesis and trichome branching [57], implying that it acts through this transcription factor in these processes. Protein-protein interactions between TCP15 and MYB106 were also reported [79]. This provides a model in which TCP15 and MYB106 control trichome branching in aerial epidermis through transcriptional regulation of common target genes (Figure 2, Table S1).

Besides trichome branching, class I TCPs are required for cuticle development possibly through different mechanisms (Figure 2). On the one hand, TCP15 directly induces *SHN1*, involved in the transcriptional regulation of cutin and wax biosynthesis genes [57]. On the other hand, TCP15 directly binds and induces genes that encode cutin and wax biosynthesis enzymes. Finally, TCP15 also acts through MYB106, a regulator of cuticle biosynthesis [57].

*TCP* genes have also been involved in the development of cotton fibers, specialized trichome cells that grow from the seed coat. Silencing of a class I TCP, named GbTCP, causes a decrease in fiber length and quality, possibly acting on the biosynthesis of jasmonic acid (JA) [23]. Another class I TCP, GhTCP14, was also proposed as a regulator of cotton fiber development through the regulation of auxin responses [80]. Manipulation of class I TCP function may then be useful to modify the properties of cotton fibers for industrial applications. In addition, AaTCP15 and AaTCP14 promote the biosynthesis of artemisin, a sesquiterpene lactone widely used in malaria treatment, in the glandular trichomes of *Artemisia annua.* These TCPs interact with AaORA, another positive regulator of artemisin biosynthesis, to synergistically activate the expression of artemisin biosynthesis genes *DBR2* and *ALDH1* in response to JA and ABA signaling [81,82], providing an interesting area of future research to explore the nexus between TCP genes, hormones and environmental parameters in the biosynthesis of specialized metabolites in plants.

In summary, TCP14 and TCP15, and probably other class I TCPs, are coordinators of aerial epidermal development and specialization. Considering the important role of the epidermis in plant defense and interactions with the environment, it would be interesting to analyze how changes in the function of the TCPs affect these processes. In addition, a possible role of the TCPs in the development of the epidermal tissue of roots, for instance in the development of root hairs, is worth studying. In this sense, it was reported that overexpression of cotton class I TCPs in *Arabidopsis* enhances root hair initiation and elongation [23,80].

### 2.4. Role of Class I TCPs in Flowering

The function of class I TCPs has also been related with the regulation of flowering time in *Arabidopsis*. TCP7, TCP14 and TCP15 positively modulate flowering through direct induction of the flowering time integrator SOC1 [60,61] (Figure 2). At least for TCP7, it was reported that its interaction with NF-Y transcription factors increases its binding efficiency to the *SOC1* promoter [61]. Notably, NF-Ys and class I TCPs were reported to mediate the effect of GA on flowering time regulation and *SOC1* expression [60,83]. In addition, TCP7 interacts with CONSTANS (CO) [61], which acts upstream of SOC1 through the regulation of *FT* expression, and several class I TCPs were shown to interact with FT [84], raising the possibility that class I TCPs exert their effects at different levels of the regulatory cascade related to flowering. In this sense, it was reported that class I TCPs induce the expression of CIN TCPs (class II) through SOC1-dependent repression of *miR319* expression [60]. Since these class II TCPs induce flowering acting on *CO* and *FT* [85,86], a regulatory loop involving these flowering time regulators has also been established.

To add complexity to the system, other class I TCPs, like TCP20, TCP22 and TCP23, were reported as negative regulators of flowering [87,88] (Figure 2). The effect of TCP20 and TCP22 on flowering seems to be related to their role in the regulation of the circadian clock component *CCA1* [88], although other mechanisms of action cannot be discarded. Notably, TCP22 and TCP23 also interact with FT and NF-Ys, like the flowering time activators TCP7, TCP14 and TCP15 [61,84]. Then, it would be interesting to investigate the effect of the interaction of the different class I TCPs with the flowering master regulator FT and how this interaction affects the expression of FT target genes. In addition, overexpression of TCP8 was reported to cause either delayed or early flowering [89,90]. Delayed flowering was related to a role of TCP8 in an FLC-dependent pathway [89], but its mechanism of action in this pathway is unknown. On the other hand, overexpression of TCP8 was found to cause an increase in the expression of the flowering time regulators *FT* and *SOC1*, while opposite changes were observed in a *tcp8* loss-of-function mutant [90]. This behavior is reminiscent of the one observed for TCP7, TCP14 and TCP15, suggesting that TCP8 may positively regulate flowering through a similar mechanism. In fact, it was observed that TCP8 directly activates the *SOC1* promoter and this activation is inhibited by co-expression of TCP23. Notably, it was reported that the different behavior of TCP8 and TCP23 is due to differences in the N-terminal portion located upstream of the TCP domain [90], raising the possibility that this region is a source of functional specificity among class I TCPs. 

In summary, as mentioned before for the regulation of cell proliferation and endoreplication, different class I TCPs seem to have opposing roles in the regulation of flowering. While a detailed molecular mechanism was reported for those TCPs that activate flowering, how other class I TCPs exert a negative regulation on this process is less clear. Whether the TCPs affect similar target genes in an opposite manner or act at a different level is worthy of investigation to fully understand their mode of action. It is noteworthy that TCP8 seems to behave like TCP14 and TCP15 during the growth of the inflorescence stem [51], regulation of plant immunity [91,92,93], and possibly endoreplication [54], while it was reported to behave distinctly during the regulation of flowering [89,90]. In a similar way, TCP7 acts through a similar mechanism as TCP14 and TCP15 during flowering, but exerts an apparently opposite effect on cell proliferation and endoreplication [49,52,53,54,57] (Figure 2). Clearly, further work is needed to understand the molecular nature of these apparent incoherent behaviors of class I TCPs. 

### 2.5. Role of Class I TCPs in Response to Light and Temperature

Increasing evidence links the function of class I TCPs with responses to changes in environmental conditions. *Arabidopsis* loss-of-function mutants in *TCP14* and *TCP15*, for instance, show increased anthocyanin accumulation in response to irradiation with high light intensity [94]. Notably, this effect, as well as the negative effect on anthocyanin accumulation of the overexpression of *TCP15*, is lost after a prolonged exposure to high-light conditions. Class I TCPs contain a redox-active conserved Cys at position 20 of the TCP domain that was reported as a control point of their DNA binding activity [95]. Since overexpression of a mutated form of TCP15 in which this Cys was changed to Ser causes a stable repression of anthocyanin accumulation, it was proposed that TCP15 is inhibited by oxidation during prolonged exposure to high irradiation conditions [94] (Figure 2). Whether similar redox changes participate in the regulation of the activity of class I TCPs during other processes in unknown.

TCP15 also participates in responses to light associated with de-etiolation. During this process, TCP15 associates with the transcription factor GLK1 [96] to induce cotyledon opening and expansion, as well as the induction of genes encoding components of the photosynthetic apparatus [66] (Figure 2, Table S1). The complex between both proteins seems to be required for a more efficient activation of genes involved in cell expansion, like *SAUR* and *EXPANSIN* genes, which contain TCP target sites in their promoters, and photosynthetic genes, which are primarily GLK1 targets. It was proposed that this mechanism ensures the coordination of cotyledon growth with the development of the photosynthetic machinery, two processes that take place during de-etiolation [66]. A role of TCP14 during de-etiolation was also proposed [97]. TCP14 induces the expression of two ELIP proteins, putatively involved in protecting the photosynthetic apparatus from damage caused by excess accumulation of free chlorophyll after illumination of etiolated plants. The function of TCP14 is counteracted in darkness by the DnaJ-like zinc finger domain-containing protein ORANGE, which interacts with TCP14 and inhibits its transactivation activity [97] (Figure 2, Table S1).

In addition, TCP15 induces cell expansion during the growth of cotyledons and petioles in response to an increase in ambient temperature [62]. During this process, TCP15 integrates into the regulatory module represented by the transcription factor PIF4 [98] through protein-protein interactions (Figure 2, Table S1). Both transcription factors target a similar group of genes with PIF4 and TCP target sites located in similar regions of the promoters. The presence of TCP15 would enhance the binding of PIF4 to these genes, thus improving their induction after a rise in ambient temperature [62]. 

Another *Arabidopsis* class I TCP, TCP22, seems to mediate the response of the circadian clock to changes in blue light conditions [99]. TCP22 interacts with the blue light receptor CRY2 and, upon illumination with blue light, it is recruited to nuclear bodies (photobodies) where it participates in the activation of the circadian clock gene *CCA1* (Table S1). The dynamics of photobody formation and disassembly would be regulated through phosphorylation of TCP22 by the protein kinase PPK1, which also forms part of the photobodies [99] (Table S1). Interestingly, TCP8, TCP14 and TCP15 are also localized to nuclear bodies after interaction with the SUMO conjugation enzyme SCE1 [100], TCP15 is incorporated into nuclear speckles formed by PIF4 upon interaction with this transcription factor [62], and TCP19 and TCP20 interact in nuclear speckles with the Pseudo-Response Regulator transcription factor PRR2 [101]. Thus, interaction of class I TCPs with different nuclear components would not only target the TCPs to specific gene promoters, but also to defined nuclear compartments engaged in the execution of different transcriptional programs. Moreover, different post-transcriptional modifications of the TCPs would affect the dynamics of the processes in which they are involved.

### 2.6. Role of Class I TCPs in Nitrate and Copper Homeostasis

Nitrogen is an essential plant component and its acquisition is crucial for growth and development. Plant roots respond to local levels of nitrate by adjusting gene expression and lateral root growth. In this process, TCP20 is a key regulator of the systemic signaling pathway that controls root system architecture and stem cell dynamics in *Arabidopsis* [102,103]. TCP20 binds to the promoter and activates the expression of the *NITRATE TRANSPORTER 1.1* (*NRT1.1*) gene, which encodes a protein acting both as an auxin transporter and a nitrate sensor, thus affecting the systemic signaling pathway that directs lateral root development during nitrate foraging [103]. Moreover, under nitrate starvation, TCP20 also interacts with NIN-like transcription factors NLP6 and NLP7, controlling the expression of key nitrate-responsive genes and regulating the expression of the cell cycle gene *CYCB1;1* and cell division in the root meristem [103] (Table S1). In addition, TCP20 interacts with the transcription factor HOMOLOG OF BRASSINOSTEROID ENHANCED EXPRESSION 2 INTERACTING WITH IBH1 (HBI1) and these two factors synergistically regulate root development in response to heterogeneous nitrate supply (Figure 2, Table S1). In roots under nitrate starvation, HBI1 and TCP20 induce genes encoding C-TERMINALLY ENCODED PEPTIDES (CEPs), involved in systemic nitrate signaling [104]. This leads to the upregulation of the nitrate transporter gene *NRT2.1* in nitrate-rich regions, which allows adjustment of root development to improve nitrate uptake [104]. It was also suggested that TCP20 and HBI1 might have a role as integrators of nitrogen and cytokinin (CK) signaling through the formation of a transcriptional complex that regulates the expression of type-A response regulators so as to amplify CK signals [104]. This is in agreement with the fact that nitrate-supplied roots produce high CK levels.

Copper (Cu), on the other hand, is an essential micronutrient for most organisms as it is involved as a cofactor in biological processes, including respiration, photosynthesis, and protection against oxidative stress. TCP16 has been implicated in the expression of the intracellular copper transporter COPT3 in *Arabidopsis*. Particularly, it binds to the *COPT3* promoter in vitro and downregulates its expression [105]. In plants with modified levels of TCP16, both the Cu content and the expression of certain markers of Cu status are altered, leading to changes in the sensitivity to limited or excessive copper availability [105]. Moreover, pollen morphology is affected in plants with altered levels of COPT3 and TCP16, in agreement with their high expression in these cells. This suggests that the regulatory pathway established by TCP16 and COPT3 would be important for the regulation of the copper status during pollen development in plants. 

Other *Arabidopsis* class I TCPs may also participate in the response to Cu availability. In fact, plants with increased expression of TCP14, TCP16, TCP19, TCP20, and TCP22 show a short-root phenotype under Cu deficiency conditions [105]. In addition, differential expression of several TCP members was observed in a global expression analysis of plants with altered levels of the copper transporter COPT2 [106]. Additional studies are required to better understand the roles of these and other TCPs in mineral uptake.

### 2.7. Role of Class I TCPs in Immunity

**Figure 3:**
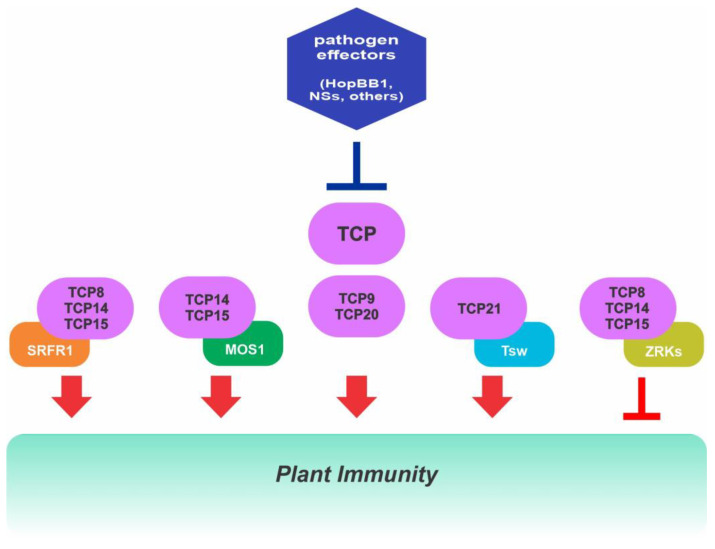
Class I TCP transcription factors play key functions in plant immunity against pathogens acting through different signaling networks. Class I TCPs from *Arabidopsis* interact with different proteins to modulate gene expression during pathogen infection and, in turn, are targeted by different pathogen effectors that affect their activity. See text for details.

In addition to regulating developmental processes, accumulating experimental evidence indicates that TCPs play key functions in plant immunity against pathogens from different kingdoms of life and through different signaling networks. TCPs are involved in the effector-triggered immunity (ETI) activated by the pathogenic bacterium *Pseudomonas syringae* in *Arabidopsis* [91,107,108,109] and a subset of class I TCPs have been shown to interact with the negative regulator of ETI SUPPRESSOR OF rps4-RLD1 (SRFR1), facilitating plant disease resistance [91]. Moreover, TCP15 and its homologues interact with MODIFIER OF snc1-1 (MOS1) to modulate plant immunity via affecting the expression of immunity genes, as the plant immune receptor gene *SNC1*, as well as several cell-cycle genes that impact immunity [53]. TCPs also participate in governing plant immunity in response to changes in ambient temperature through the formation of complexes with HOPZ-ETI-DEFICIENT 1 (ZED1)-related kinases (ZRKs) that inhibit SNC1 transcription [110] (Figure 3, Table S1). In addition, several class I TCPs act as positive regulators of EFR (EF-Tu receptor)-dependent PAMP-triggered immunity (PTI) [93], function redundantly to establish systemic acquired resistance (SAR) [92] and constitute a regulatory node for communication between JA and salicylic acid (SA) signaling during the immune defense. For instance, TCP8 and TCP9 positively regulate the expression of *ISOCHORISMATE SYNTHASE 1* (*ICS1*), which is responsible for pathogen-induced SA biosynthesis, upon pathogen infection [111]. Moreover, TCP8 interacts with most transcription factors involved in the regulation of *ICS1*, suggesting that TCP proteins may act as orchestrators to regulate the expression of *ICS1* during pathogen infection. Meanwhile, TCP9 and TCP20 negatively regulate JA synthesis by directly binding to the JA biosynthesis gene *LOX2* [112] and TCP14 regulates the plant immune system by repressing the JA signaling pathway [113]. In soybean and eggplant, GmTCP19-Like (GmTCP19L) and SmTCP7a modulate resistance to *Phytophthora sojae* [22] and *R. solanacearum* [114], respectively, but the molecular mechanisms involved are still unknown. A recent report indicated that TCP9 modulates root system architectural plasticity in response to infections by the endoparasitic cyst nematode Heterodera schachtii via reactive oxygen species (ROS)-mediated processes in Arabidopsis, establishing a novel tolerance mechanism that mitigates the impact of biotic stress rather than targeting the causal agent [115].

Recently, TCP transcription factors were proposed as targeted effector hubs [116], since they are targets of diverse secreted effector proteins from pathogens from different kingdoms, as bacteria, fungi and herbivorous arthropods [107,108,113,117,118]. Interestingly, class I TCPs are targeted more often by effectors than class II members, with TCP14 showing the highest number of interactions with effectors among all known effector hubs [118]. The rationale behind why TCPs are targeted so prominently is still unclear. Effectors might target different TCPs to modulate the same processes at different levels, thereby inhibiting TCP redundant activities. Interestingly, many of the identified TCP effector hubs seem to play a coordinated role in SAR and SA biosynthesis. Effectors that interfere with these processes can negatively affect plant defense responses. For instance, the Pseudomonas HopBB1 effector targets TCP14 to antagonistically suppress SA and increase plant susceptibility to a hemibiotrophic pathogen [113] (Figure 3, Table S1). Pathogens are found to manipulate progression of the cell-cycle in plants for their own propagation, while plants appear to modulate their cell-cycle to enhance resistance in both PTI and ETI. Recent evidence also indicates that a viral pathogen effector, NSs, targets TCP21 and related class I TCPs to manipulate hormonal responses in pepper [119] (Figure 3, Table S1). TCP21 serves as a bridge between NSs and several hormone receptors, thus affecting the interaction of the receptors with the corresponding repressors and inhibiting their degradation, which has a negative effect on plant immunity. In addition, TCP21 also bridges the interaction of NSs with the immune receptor Tsw, facilitating effector recognition and defense responses [119] (Table S1). This shows that plants may also guard effector targeted TCPs to detect pathogen infection and trigger an efficient immune response. So, increasing evidence indicates that TCPs serve as a bridge to connect developmental processes with plant immunity and the mechanisms by which effectors affect TCPs to facilitate proliferation of pathogens are yet to be fully uncovered.

### 2.8. Role of Class I TCPs in Abiotic Stress Responses

Several reports indicate that the expression of class I TCPs is upregulated under abiotic stress conditions, as drought and salinity, and after ABA treatment in a range of plant species, such as rice, maize, cowpea, potato, and moso bamboo [120,121,122,123,124]. Moreover, overexpression or ectopic expression of class I TCPs (as PeTCP10, VuTCP9, and OsPCF2) enhances tolerance to drought and/or salt and ABA sensitivity [19,120,121,122,124]. Overexpression of rice OsTCP19 in *Arabidopsis* causes the upregulation of *ABI3* and *ABI4* [19] and PCF2 positively regulates the expression of the vacuolar K^+^-Na^+^/H^+^ antiporter *NHX1* in rice [124], whereas PeTCP10 directly binds to the promoter of the ABA-responsive gene *BT2* [120], suggesting that class I TCP members exert regulatory functions in drought and salinity stress tolerance acting at different levels of the ABA signaling cascade. Notably, although these findings reveal TCPs as positive regulators of ABA-mediated abiotic stress tolerance, a negative effect on ABA biosynthesis and signaling was reported for a group of class I TCP members. In apple, MdTCP46 reduces the expression of MdABI5 and its transcriptional activity by interfering with its binding to *MdEM6* and *MdRD29A* target genes, and thus reducing the ABA-dependent drought response [24] (Table S1). GhTCP19 inhibits ABA biosynthesis directly repressing *GhNCED* to promote corm dormancy release in gladiolus [125] and TCP14 inhibits the expression of the ABA biosynthesis gene *ABA1* to promote seed germination in *Arabidopsis* [71,72]. Meanwhile, StTCP15 negatively affects ABA content in potato tubers to promote tuber dormancy release [123]. These results point out that several class I TCPs would modulate different aspects of plant growth and development through the inhibition of ABA-related pathways at multiple levels. Since ABA is an inhibitor of processes promoted by TCPs (e.g., plant growth and reproduction and cell division and elongation) a mutual repression between class I TCP activity and ABA signaling pathways is expected. According to this, the expression of *MdTCP46* is repressed by ABA and drought conditions in apple [24]. However, despite increasing reports in this area, the molecular network connecting TCP function with ABA-dependent signaling pathways are far from being understood. In the future, elucidating the molecular regulatory networks in which the different members of the TCP family participate during the response of plants to abiotic stress and ABA will allow us to increase our knowledge about the regulatory mechanisms balancing plant growth with stress responses and to explore the potential of the TCPs for plant improvement. 

## 3. Modulation of Class I TCP Protein Activity

Transcription factors are considered as master regulators involved in important plant responses associated with genetic reprogramming and it is well acknowledged that their activity need to be highly and finely tuned. Thus, different regulatory mechanisms such as post-translational modifications, protein-protein interactions, protein degradation or stabilization, but also protein relocalization can be considered. These interactions are highly dynamic and might affect positively or negatively the stability of transcription factors, modify their DNA binding activities and have consequences on the expression of target genes. A growing body of evidence shows the existence of a precise regulation of the activity of TCP proteins through the modulation of protein stability, DNA binding capacity and subcellular localization. For example, class I TCP protein levels are affected by proteasome-dependent degradation mechanisms [113]. DA1, DAR1 and DAR2 peptidases cleave TCP14, TCP15 and TCP22, leading to their inactivation and destabilization to limit cell proliferation in *Arabidopsis* [52,126], whereas SPINDLY (SPY) interacts with TCP14 and TCP15 preventing their proteolysis by the 26S proteasome [127,128] (Figure 2, Table S1). In the absence of SPY, degradation of the TCPs is governed by interaction with F-box proteins of the KISS ME DEADLY family, negative regulators of CK signaling [129]. Recently, SPY was demonstrated to be an O-fucosyltransferase that modifies a number of proteins [130,131,132]. Inhibition of class I TCP proteolysis by SPY promotes CK responses in developing *Arabidopsis* leaves and flowers and the catalytic domain of SPY was identified as essential for TCP activity. However, whether SPY indeed O-fucosylates TCPs for stabilization and the mechanism by which SPY affects TCPs accumulation or stability are critical questions to answer. In addition, TCPs were identified as downstream interacting partners of mitogen-activated protein kinases (MAPKs) and PHOTOREGULATORY PROTEIN KINASES (PPKs) and evidence of direct O-GlcNAc modification and phosphorylation near the N-terminal of class I TCPs was reported [55,99,110,123,133,134]. However, the extent or roles of these modifications in the activity of TCPs are still unknown. Future studies are needed to establish the functional links between O-GlcNAcylation and phosphorylation of TCPs and plant growth regulation. 

### 3.1. Interaction with Non-TCP Transcriptional Regulators

TCP proteins can function either as transcriptional activators or repressors and act through recruitment of specific non-TCP proteins by protein-protein interactions. The formation of these complexes can lead to an increase in the transcriptional activity of TCPs or exert an inhibitory or antagonistic effect, depending on the specific proteins that TCPs interact with. In some cases, a synergistic or cooperative effect on the transcriptional activity of interacting proteins was observed [53,55,61,66,82,88,92,99,104], in others, binding to target gene promoters is possible or enhanced by protein-protein complex formation [53,66,92,99] or, even if TCPs can bind to promoters of target genes, they function as transcriptional activators only if they interact with a partner [66,99] (Table S1). On the other hand, there are protein partners that inhibit the transcriptional activity of TCPs, as for example ORANGE, ERF4 and DELLA proteins [56,78,97] (Figure 2, Table S1). DELLA proteins interact with the DNA binding motif of TCPs, sequestering them into inactive complexes unable to bind target genes [56], whereas the transcriptional repressor ERF4 inhibits the ability of *Arabidopsis* TCP15 to activate transcription by interaction with other regions of the protein [78]. In addition, TCP15 acts as a working partner with ERF4 to antagonistically regulate the expression of their targets [78]. 

Although most of the class I TCP proteins have been reported as transcriptional activators, a number of reports indicate that they can also act as repressors. TCP16 and TCP21/CHE1 from *Arabidopsis*, PpTCP20 from peach, and GhTCP19 from cotton repress the expression of their target genes [26,125,135,136], MdTCP46 from apple blocks the binding of a transcriptional activator to its target genes, thereby negatively regulating their expression [24], and some TCPs interact with transcriptional repressors [137]. Even more, different class I TCPs can act as activators or repressors of the same target gene, as *CCA1*, which is activated by TCP20 and TCP22 and repressed by TCP21/CHE in *Arabidopsis* [99,136]. All this indicates that the vast capacity of TCPs to form complexes with different types of proteins provides a flexible mechanism to regulate growth and development in plants.

### 3.2. Class I TCPs in Redox Signaling

Interestingly, class I TCP transcription factor activity is regulated in a redox-dependent manner. The DNA binding capacity of class I TCPs was shown to be redox-modulated through the oxidation of a highly conserved cysteine residue localized at the beginning of helix I of the TCP domain [94]. Oxidizing conditions lead to the formation of an intermolecular disulfide bond between two TCP domains that would affect TCP dimer conformation, such that DNA binding and transcriptional regulation of TCP target genes is no longer possible [95]. This indicates that class I TCPs can act as sensors of altered redox conditions, as imbalanced H_2_O_2_ levels generated in response to environmental changes. Recently, a similar redox-dependent DNA interaction was reported for the only class I TCP from the liverwort *Marchantia polymorpha*, MpTCP1 [18]. Given the presence of a single conserved cysteine residue in charophycean algae and land plants, its presence might already have contributed to sensing and responding to redox changes in water-living algae and then in early diverging land plants. 

Evidence has been gathered that TCPs are involved in ROS-mediated processes during stress-induced adaptive plant growth responses. For example, class I TCPs repress anthocyanin biosynthesis in *Arabidopsis*, but a prolonged exposure to high light intensity leads to redox inactivation of TCPs, de-repression of anthocyanin synthesis, and a protective response [94] (Figure 2). MpTCP1 senses ROS levels and affects the expression of several enzymes involved in ROS metabolism, mediating adaptive responses to heat stress [13]. In *Arabidopsis*, TCP9 modulates ROS homeostasis in response to nematode infection [115] and class I TCP double and triple mutants exhibit enhanced ROS production [78]. It was also reported that expression of moso bamboo PeTCP10 increases drought tolerance in transgenic *Arabidopsis* via ROS-regulated root growth [120,121].

In summary, several reports have indicated that class I TCPs are targets as well as modulators of changes in cellular redox homeostasis, suggesting that they may act as sensors during the response to internal and environmental conditions that affect the redox status of the cell. In this sense, it has been proposed that lower ROS levels would activate TCPs to regulate the expression of cell cycle-related genes and that JA, glutathione and TCPs might form a molecular network that controls redox regulation of the cell cycle in plants [138,139]. However, further research is needed to unravel how TCPs function, transcriptional regulation of ROS-related processes, ROS sensitivity and accumulation, and the stress-induced growth response pathways, are all connected. In addition, the effect of redox regulation of the TCPs on their interaction with non-TCP proteins, their subcellular and subnuclear localization and other post-translational modifications that may affect their activity or stability is still an open question. 

### 3.3. Subcellular Distribution of Class I TCPs 

TCP proteins were identified as nuclear proteins that can localize into substructures or subdomains in the nucleus. Interestingly, several reports indicate that nuclear distribution of TCPs differs between members and also their location is differently affected by interacting proteins. TCP14 was detected exclusively in nuclear bodies, whereas TCP8 and TCP15 homodimers were shown to localize in nuclear bodies and the nucleoplasm, respectively, in *Arabidopsis* [13,91,100,113]. Nuclear aggregate formation was linked to the presence of an intrinsically disordered region in the C-terminus of TCP8 [13] and requires an intact DNA-binding ability in TCP14 [113]. Interestingly, a relocation of TCP8 and TCP15 was observed upon interaction with some partners. For example, TCP8 interacts with SRFR1 in nuclear foci, but TCP8-PNM1 complexes were detected in the nucleoplasm in BiFC assays [91,140]. Furthermore, TCP8 nuclear localization seems to be affected by interaction with BZR1 [141]. As observed with homodimers, TCP15 was detected in the nucleoplasm when interacting with SRFR1, MYB106 and GLK1 [66,79,91]. However, interaction with PIF4 relocates TCP15 to nuclear speckles [62]. In addition, TCP19 and TCP20 mediate the localization of PRR2 in Cajal bodies and nuclear speckles [101], evidencing that sub-nuclear localization of TCP proteins is dependent on their interacting partners. According to the literature, accumulation of nuclear factors in distinct nuclear bodies may help to generate a high local concentration of components. This could ultimately either enhance or decrease the biological function of such proteins. This sub-nuclear compartmentalization process might also contribute to modifying protein behavior and to the regulation of their stability or activity. In this sense, TCP14 nuclear bodies are recruited to JAZ3-degradation bodies by the effector protein HopBB1 from *Pseudomonas syringae* [113]. In addition, TCP8, TCP14 and TCP15 are redistributed into nuclear foci or speckles when bound to the SUMO conjugation enzyme SCE1, suggesting that these TCP foci are sites for SUMO conjugation of TCPs [100]. A recent discovery indicates that TCP1 from *Marchantia polymorpha* (MpTCP1) acts as a transcriptional repressor through its ability to form protein speckles in the nucleus and thereby physically block access to the chromatin [142]. The nature of these subnuclear localizations has yet to be explored, but could be sites of suppression, enhanced activation, or both, at multiple genetic loci. As also mentioned above, recruitment to specific sites may be involved in the degradation or post-translational modification of the TCPs. Future work should focus on the conditions or factors that modulate the sub-nuclear localization patterns of class I TCP members and the biological relevance of the formation of these TCP-containing nuclear bodies. In addition, although TCPs are primarily detected in the nucleus, different localizations of TCP-containing protein-protein complexes were observed depending on environmental conditions [102]. These reports evidence an additional level of TCP activity modulation depending not only on their interacting partners but also on cellular or environmental conditions.

## 4. Concluding Remarks and Perspectives

Class I TCP transcription factors play central roles in numerous plant growth and developmental processes through the direct control of cell cycle progression and cell elongation and the regulation of biosynthesis and signaling pathways of many plant hormones. They are crucial for the response of plants to variations in environmental cues, as nutrients, light, and temperature, and unraveling how environmental inputs influence TCP class I-mediated growth control is becoming an area of great interest.

Most studies have focused on the model system, *Arabidopsis*, but in recent years, TCP class I functions are beginning to be elucidated in non-model systems as well. It has been assumed that the class I TCP members act in a semi-redundant fashion, but evidence has accumulated indicating that different members act at different levels of molecular pathways. Moreover, opposite effects were reported in certain processes, which explains the maintenance of multiple members in the TCP family in the different species. In the future, the functional characterization of the individual class I TCP members will be crucial to shed light on the specific roles of these proteins. 

The complex regulatory network of class I TCP transcription factors can be explained by their unique mode of DNA binding, that would confer broad specificity for a range of DNA sequences, and by their ability to interact with multiple plant proteins, leading to an increased transcriptional activity or exerting an inhibitory or antagonistic effect. This places them in central positions in plant signaling cascades, and marks them as attractive effector targets for diverse pathogens and the response to environmental conditions and abiotic stress, suggesting that class I TCPs are interesting tools to optimize plant characteristics and the response to environmental challenges. Furthermore, elucidating how class I TCPs, and their partners, integrate into redox signaling pathways to modulate redox signal-derived growth and stress responses will be an emerging field of study.

## Data Availability

Not applicable.

## Supplementary Materials

The supporting information can be downloaded at: https://www.mdpi.com/article/10.3390/ijms24065437/s1.
