# Supplementary material for: Physiological Roles and Mechanisms of Action of Class I TCP Transcription Factors
Source: Int J Mol Sci. 2023 Mar 12;24(6):5437. doi: 10.3390/ijms24065437 (PMC10049435; doi:10.3390/ijms24065437)
Supplement: Supplementary file 1 [file ijms-24-05437-s001.zip › ijms-2158672-supplementary.pdf]

Table S1. A summary of processes affected by class I TCPs and their interacting partners. See text for details. Abbreviations are as mentioned in text.

TCP	Interacting protein	Type	Effect of the interaction	Process	Organism	Reference
AtTCP14<br>AtTCP15<br>AtTCP22	DA1<br>DAR1<br>DAR2	ubiquitin receptor	TCP degradation	cell proliferation	*Arabidopsis thaliana*	(52)<br>(127)
AtTCP15	ERF4	transcription factor	inhibits TCP transcriptional activity	cell cycle regulation	*Arabidopsis thaliana*	(78)
AtTCP15	PIF4	transcription factor	increases binding to target genes	cell expansion, thermomorphogenesis	*Arabidopsis thaliana*	(62)
AtTCP14<br>AtTCP15	DELLAs	transcriptional repressors	inhibits TCP transcriptional activity	germination, stem growth	*Arabidopsis thaliana*	(51) (56)
AtTCP14	DOF6	transcription factor	non tested	germination	*Arabidopsis thaliana*	(72)
AtTCP14	MPK8	MAP kinase	increases TCP transcriptional activity	germination	*Arabidopsis thaliana*	(55)
AtTCP15	GLK1	transcription factor	increases binding to target genes	cotyledon development	*Arabidopsis thaliana*	(66)
AtTCP14	ORANGE	DnaJ-like zinc finger domain protein	inhibits TCP transcriptional activity	greening	*Arabidopsis thaliana*	(97)
AtTCP15	MYB106	transcription factor	Non tested	epidermis development	*Arabidopsis thaliana*	(79)
AtTCP7	NF-Ys	transcription factor	increases binding to target genes	flowering	*Arabidopsis thaliana*	(61)
AtTCP7	CO	transcription factor	non tested	flowering	*Arabidopsis thaliana*	(61)
AtTCP7<br>AtTCP14<br>AtTCP15<br>AtTCP22<br>AtTCP23	FT	transcription factor	non tested	flowering	*Arabidopsis thaliana*	(84)
AtTCP22<br>AtTCP23	NF-Ys	transcription factor	non tested	flowering	*Arabidopsis thaliana*	(83)
AtTCP22	CRY2	blue light receptor	increases binding to target genes	circadian clock regulation	*Arabidopsis thaliana*	(99)
AtTCP22	PPK1	protein kinase	increases binding to target genes	circadian clock regulation	*Arabidopsis thaliana*	(99)
AtTCP20<br>AtTCP21<br>AtTCP22	LWD1	WD repeat-containing protein	increases transcriptional activity	circadian clock regulation	*Arabidopsis thaliana*	(88)<br>(99)
AtTCP20	NLP6<br>NLP7	transcription factor	increases TCP transcriptional activity	nitrate signaling	*Arabidopsis thaliana*	(102)
AtTCP20	HBI1	transcription factor	increases TCP transcriptional activity	nitrate signaling	*Arabidopsis thaliana*	(104)
AtTCP14<br>AtTCP15	SPINDLY	O-fucosyltransferase	prevents TCP proteolysis	CK signaling	*Arabidopsis thaliana*	(128)<br>(129)<br>(130)
MdTCP46	MdABI5	transcription factor	represses MdABI5 binding to target genes	ABA response	*Malus domestica*	(24)
AaTCP14<br>AaTCP15	AaORA	transcription factor	increases TCP transcriptional activity	artemisinin biosynthesis	*Artemisia Annua*	(81)<br>(82)

AaTCP14	AaJAZ8	JAZ protein	represses TCP transcriptional activity	artemisinin biosynthesis	*Artemisia Annua*	(82)
AtTCP14<br>AtTCP15	MOS1	HLA-B ASSOCIATED TRANSCRIPT2 (BAT2) domain-containing protein	increases binding to TCP target genes	plant immunity	*Arabidopsis thaliana*	(53)
AtTCP15	NPR1	transcriptional cofactor	increases binding to target genes	plant immunity	*Arabidopsis thaliana*	(92)
AtTCP8<br>AtTCP14<br>AtTCP15	SRFR1	tetratricopeptide (TPR) protein	non tested	plant immunity	*Arabidopsis thaliana*	(107)
AtTCP8<br>AtTCP14<br>AtTCP15	ZRKs	kinase	non tested	plant immunity	*Arabidopsis thaliana*	(111)
AtTCP14	HopBB1	bacterial type III effector	TCP degradation	plant immunity	*Arabidopsis thaliana*	(114)
AtTCP21	NSs	viral pathogen effector	inhibits phytohormone receptors function	plant immunity	*Arabidopsis thaliana*	(120)
AtTCP21	Tsw	immune receptor	immune effector recognition	plant immunity	*Arabidopsis thaliana*	(120)
